# Continual Decline in Azole Susceptibility Rates in *Candida tropicalis* Over a 9-Year Period in China

**DOI:** 10.3389/fmicb.2021.702839

**Published:** 2021-07-09

**Authors:** Yao Wang, Xin Fan, He Wang, Timothy Kudinha, Ya-Ning Mei, Fang Ni, Yu-Hong Pan, Lan-Mei Gao, Hui Xu, Hai-Shen Kong, Qing Yang, Wei-Ping Wang, Hai-Yan Xi, Yan-Ping Luo, Li-Yan Ye, Meng Xiao, China Hospital Invasive Fungal Surveillance Net (CHIF-NET) Study Group, Zi-Yong Sun

**Affiliations:** Tongji Hospital, Tongji Medical College of Huazhong University of Science and Technology; Peking Union Medical College Hospital, Chinese Academy of Medical Sciences; West China Hospital, Sichuan University; Jiangsu Province Hospital; Fujian Medical University Union Hospital; First Affiliated Hospital of Zhengzhou University; First Affiliated Hospital, Sun Yat-sen University; First Affiliated Hospital, Zhejiang University School of Medicine; Jinling Hospital, Nanjing University School of Medicine; Chinese PLA General Hospital, Medical School of Chinese PLA; Sichuan Provincial People’s Hospital, Sichuan Academy of Medical Sciences; First Affiliated Hospital of Harbin Medical University; Affiliated Hospital of Qingdao University; Shandong Provincial Hospital; Sir Run Run Shaw Hospital; Peking University First Hospital; Union Hospital, Tongji Medical College, Huazhong University of Science and Technology; Xiangya Hospital, Central South University; First Affiliated Hospital of Wenzhou Medical University; Fujian Provincial Hospital; Second Affiliated Hospital Zhejiang University School of Medicine; Changhai Hospital University School of Medicine; First Affiliate Hospital of Guangzhou Medical University; Huashan Hospital, Fudan University; First Affiliated Hospital of Chongqing Medical University; Tianjin Medical University General Hospital; Tianjin First Central Hospital; Southwest Hospital of Army Medical University; Second Xiangya Hospital of Central South University; First Hospital of China Medical University; ; First Affiliated Hospital of University of Science and Technology of China; General Hospital of Ningxia Medical University; Zhejiang Provincial People’s Hospital; Air Force Medical University; First Affiliated Hospital of University of Science and Technology of China; Guangzhou First People’s Hospital; Lisui Municipal Central Hospital; First Affiliated Hospital, Kunming Medical University; First Affiliated Hospital of Guangxi Medical University; First Affiliated Hospital of Fujian Medical University; Henan Provincial People’s Hospital; People’s Hospital of Liaoning Province; Ruian People’s Hospital; Yantai Yuhuangding Hospital; People’s Hospital of Zhengzhou; Dao-Hong Zhou, Daping Hospital, Third Military Medical University; First Affiliated Hospital of Xi’an Jiaotong University; First Affiliated Hospital of Dalian Medical University; Third Attached Hospital, Sun Yat-sen University; Fourth Hospital of Harbin Medical University; First Bethune Hospital of Jilin University; Shengjing Hospital of China Medical University; Ruijin Hospital, Shanghai Jiao Tong University of Medicine; First Hospital of Jiaxing; Taizhou Hospital of Zhejiang Province; Lanzhou University Second Hospital; Ya-Lu Ren, First Affiliated Hospital of Soochow University; Second Hospital of Hebei Medical University; China-Japan Union Hospital of Jilin University; Affiliated Hospital of Guizhou Medical University; Dalian Municipal Central Hospital; WuHan No.1 Hospital; Shanghai Changzheng Hospital; Hwa Mei Hospital, University of Chinese Academy of Sciences; Jiangxi Province People’s Hospital; First Affiliated Hospital of Shandong First Medical University; Jing Zhu, Fouth Medical Center of PLA General Hospital; Second Hospital of Shanxi Medical University; Guangdong Provincial People’s Hospital, Guangdong Academy of Medical Sciences; First Affiliated Hospital of Xinjiang Medical University; Beijing Tongren Hospital, Capital Medical University; General Hospital of Lanzhou Military Region; First Hospital of Shanxi Medical University; 3201 Hospital; Shanghai General Hospital; Tangshan Gongren Hospital; Second Affiliated Hospital of Nanchang University; People’s Hospital of Xinjiang; Xin Wang, Gansu Provincial Hospital; Jiujiang No.1 People’s Hospital; Jun-Rui Wang, Affiliated Hospital of Inner Mongolia Medical University; Zhongshan Hospital Xiamen University; Hebei General Hospital; Qilu Hospital of Shandong University; 94th Hospital of Chinese PLA; Qingdao Municipal Hospital; General Hospital of Northern Theater Command; ^1^Department of Laboratory Medicine, Peking Union Medical College Hospital, Chinese Academy of Medical Sciences, Beijing, China; ^2^Beijing Key Laboratory for Mechanisms Research and Precision Diagnosis of Invasive Fungal Diseases, Peking Union Medical College Hospital, Chinese Academy of Medical Sciences, Beijing, China; ^3^State Key Laboratory of Complex Severe and Rare Diseases, Peking Union Medical College Hospital, Chinese Academy of Medical Sciences, Beijing, China; ^4^Department of Infectious Diseases and Clinical Microbiology, Beijing Chaoyang Hospital, Capital Medical University, Beijing, China; ^5^School of Biomedical Sciences, Charles Sturt University, Orange, NSW, Australia; ^6^New South Wales Health Pathology, Regional and Rural, Orange Hospital, Orange, NSW, Australia; ^7^Department of Clinical Laboratory, Jiangsu Province Hospital, Nanjing, Jiangsu, China; ^8^Department of Clinical Laboratory, Fujian Medical University Union Hospital, Fuzhou, China; ^9^Department of Clinical Laboratory, First Affiliated Hospital of Zhengzhou University, Zhengzhou, China; ^10^Department of Laboratory Medicine, First Affiliated Hospital of Zhejiang University School of Medicine, Hangzhou, China; ^11^Institute of Laboratory Medicine, Jinling Hospital, Nanjing University School of Medicine, Nanjing, China; ^12^Medical Laboratory Center, Chinese PLA General Hospital, Medical School of Chinese PLA, Beijing, China

**Keywords:** *Candida tropicalis*, antifungal susceptibility, azole, echinocandin, antifungal resistance

## Abstract

**Background:**

There have been reports of increasing azole resistance in *Candida tropicalis*, especially in the Asia-Pacific region. Here we report on the epidemiology and antifungal susceptibility of *C. tropicalis* causing invasive candidiasis in China, from a 9-year surveillance study.

**Methods:**

From August 2009 to July 2018, *C. tropicalis* isolates (*n* = 3702) were collected from 87 hospitals across China. Species identification was carried out by mass spectrometry or rDNA sequencing. Antifungal susceptibility was determined by Clinical and Laboratory Standards Institute disk diffusion (CHIF-NET10–14, *n* = 1510) or Sensititre YeastOne (CHIF-NET15–18, *n* = 2192) methods.

**Results:**

Overall, 22.2% (823/3702) of the isolates were resistant to fluconazole, with 90.4% (744/823) being cross-resistant to voriconazole. In addition, 16.9 (370/2192) and 71.7% (1572/2192) of the isolates were of non-wild-type phenotype to itraconazole and posaconazole, respectively. Over the 9 years of surveillance, the fluconazole resistance rate continued to increase, rising from 5.7 (7/122) to 31.8% (236/741), while that for voriconazole was almost the same, rising from 5.7 (7/122) to 29.1% (216/741), with no significant statistical differences across the geographic regions. However, significant difference in fluconazole resistance rate was noted between isolates cultured from blood (27.2%, 489/1799) and those from non-blood (17.6%, 334/1903) specimens (*P*-value < 0.05), and amongst isolates collected from medical wards (28.1%, 312/1110) versus intensive care units (19.6%, 214/1092) and surgical wards (17.9%, 194/1086) (Bonferroni adjusted *P*-value < 0.05). Although echinocandin resistance remained low (0.8%, 18/2192) during the surveillance period, it was observed in most administrative regions, and one-third (6/18) of these isolates were simultaneously resistant to fluconazole.

**Conclusion:**

The continual decrease in the rate of azole susceptibility among *C. tropicalis* strains has become a nationwide challenge in China, and the emergence of multi-drug resistance could pose further threats. These phenomena call for effective efforts in future interventions.

## Introduction

*Candida* species are leading fungal pathogens causing invasive fungal diseases worldwide, and can be life-threatening with notable mortality (Kullberg and Arendrup, 2015; Pappas et al., 2018). *Candida albicans* remains the predominant species implicated in invasive candidiasis (IC), and is generally susceptible to all antifungal agents, including azoles and echinocandins (Kullberg and Arendrup, 2015; Pappas et al., 2016, 2018; Perlin et al., 2017). However, a rising trend in the detection rates of non-*albicans Candida* species has been observed, mostly due to the wide use of antifungals, as many of these species are less susceptible (Perlin et al., 2017). The top three non-*albicans Candida* species most described worldwide are *Candida glabrata sensu stricto*, *Candida tropicalis*, and *Candida parapsilosis sensu stricto*, but with significant geographic variations (Kullberg and Arendrup, 2015; Perlin et al., 2017; Pappas et al., 2018). Amongst these species, *C. tropicalis* has been detected at significantly higher prevalence rates in Asia and Latin-America regions (Tan et al., 2016; Pfaller et al., 2019; Xiao et al., 2020).

To date, there are only four classes of antifungals used for IC, namely azoles, echinocandins, polyenes, and nucleoside analogs (Pappas et al., 2016). Without any other antifungal therapy alternatives, resistance to any of these antifungal classes could pose a great threat to patients (Pappas et al., 2016; Perlin et al., 2017). Previous studies have shown that antifungal resistance in *Candida* species varies across geographic regions worldwide. For *C. tropicalis*, low resistance rates to azoles have been reported in North America, Latin-America, and most European Countries (fluconazole resistant rate <5%) as per the SENTRY global surveillance program (Pfaller et al., 2019), but high rates (23.2% to fluconazole) were reported by Fernandez-Ruiz et al. (2015) in Spain. In contrast, a high azole resistance rate for *C. tropicalis* has been observed in the Asia-Pacific region, especially in mainland China and Taiwan (Fan et al., 2017; Chen et al., 2019; Pfaller et al., 2019; Xiao et al., 2020). In a recent report by Chen et al. (2019), 16.9% of *C. tropicalis* isolates collected in Taiwan were non-susceptible to fluconazole. Meanwhile, the fluconazole resistance rate of *C. tropicalis* in China mainland have exceeded 25%, with over 90% of these isolates cross-resistant to voriconazole (Wang et al., 2020; Xiao et al., 2020). In addition, echinocandin drugs including caspofungin, micafungin, and anidulafungin, have been commercially used for treatment of IC worldwide. Thus emergence of echinocandin- and multidrug-resistance in *C. tropicalis* raises further concerns for clinical management of patients (Jensen et al., 2013; Khan et al., 2018; Xiao et al., 2018; Pfaller et al., 2019; Arastehfar et al., 2020).

As growing challenges of antifungal resistance in *C. tropicalis* have been noted, it is important that continual surveillance targeting this species be implemented in all regions of China and elsewhere. The CHIF-NET study is a laboratory-based, nationwide multicenter study of invasive yeast infections, including IC in China, which was initiated in August 2009. As of July 2018, a total of 87 hospitals had participated in this program for a period of nine surveillance years (CHIF-NET10 to CHIF-NET18). Here we report essential findings on the epidemiology and antifungal susceptibility patterns of *C. tropicalis* causing IC from the CHIF-NET program. Of note and worrying, is the continual decreasing trend of azole susceptibility rate, and fluconazole non-susceptible rates, among *C. tropicalis* strains, which has risen to around 45% nationwide.

## Materials and Methods

### Study Design

During August 2009 to July 2018, a total of 87 hospitals participated in CHIF-NET program, with 79.3% (69/87) of these sites having participated for at least 3 years or longer (median duration of participation, 5 years). Inclusion and exclusion criteria for the isolates were the same as previously described (Xiao et al., 2020). Of note, in the case of multiple *C. tropicalis* isolates from one patient, only one isolate was included in the analysis. In each surveillance year, all isolates from the participating hospitals were sent to a central laboratory (Department of Laboratory Medicine, Peking Union Medical College Hospital) for confirmative identification and antifungal susceptibility testing.

### Species Identification

Isolates collected from CHIF-NET10 and CHIF-NET11 were identified by DNA sequencing of the fungal rDNA internal transcribed spacer region supplemented with D1/D2 domain of the 28S rRNA gene, as previously described (Wang et al., 2012). From CHIF-NET12 to CHIF-NET18, species identification was carried out by matrix-assisted laser desorption ionization-time of flight mass spectrometry (MALDI-TOF MS) (Vitek MS, IVD database V2.0/2.1/3.0, bioMérieux, France, CHIF-NET12 to CHIF-NET16; and Autof MS 1000, Autof Acquirer Version V2.0.18, Autobio Diagnostics, China, CHIF-NET17 to CHIF-NET18). For any isolates that could not be identified or with uncertain identification results to species level by MALDI-TOF MS, rDNA sequencing was performed as “gold standard” (Xiao et al., 2020).

### Antifungal Susceptibility Testing

Susceptibility to fluconazole and voriconazole was determined using the Clinical and Laboratory Standards Institute (CLSI) disk diffusion method (disks purchased from Oxoid, Thermo Fisher Scientific, Hampshire, United Kingdom) for isolates collected from CHIF-NET10 to CHIF-NET14 (CLSI, 2020b). From CHIF-NET15 to CHIF-NET18, the *in vitro* susceptibility to nine antifungal agents, including fluconazole, voriconazole, itraconazole, posaconazole, caspofungin, micafungin, anidulafungin, amphotericin B, and 5-flucytosine, was determined using Sensititre YeastOne^TM^ YO10 methodology (Thermo Scientific, Cleveland, OH, United States) following manufacturer’s instructions. Current available clinical breakpoints (CBPs) or epidemiological cut-off values (ECVs) were used for interpretation of susceptibility results (Fan et al., 2017; CLSI, 2020a,b). *Candida parapsilosis* ATCC 22019 and *Candida krusei* ATCC 6258 were used for quality control for each run of susceptibility testing, and all quality control results were within published ranges.

### Susceptibility Interpretation and Statistical Analysis

Disk diffusion diameter and minimum inhibitory concentration (MIC) results of fluconazole and voriconazole, and MICs of three echinocandin agents were interpreted as per the latest CLSI CBPs (CLSI, 2020b). In addition, ECVs were used for interpretation of itraconazole, posaconazole, amphotericin B (CLSI, 2020a), and 5-flucytocine (Xiao et al., 2020).

For statistical analyses, Chi-square test was performed using IBM SPSS software (version 22.0; IBM SPSS Inc., Armonk, NY, United States), and Bonferroni *post hoc* test was carried out for multiple comparisons when necessary. A *P*-value (or Bonferroni adjusted *P*-value) of <0.05 was considered significant.

## Results

### Demography Characters

A total of 3702 *C. tropicalis* isolates were collected over a period of 9 years from 87 different hospitals in China. In each surveillance year, 122–741 *C. tropicalis* isolates were identified. The number of participating hospitals and isolates collected in each year are shown in Supplementary Figure 1. For patients with IC due to *C. tropicalis*, the majority (64.9%; 2404/3702) were male. Patient ages ranged from 0 to 103 years (median, 56; interquartile, 41–68).

### Antifungal Susceptibilities in General

During the first five surveillance years of the CHIF-NET program (CHIF-NET10–14), only susceptibility to fluconazole and voriconazole was performed. However, from CHIF-NET15–18, susceptibilities to nine antifungal drugs were performed (Table 1).

**TABLE 1:** Distribution and antifungal susceptibility of *C. tropicalis* by clinical services and specimen types.

**Characters**	**No. of isolates (%)**		**Antifungal susceptibility (%)**																	
	**CHIF-NET 10–18**	**CHIF-NET 15–18**	**Fluconazole^a^**		**Voriconazole^a^**		**Itraconazole^b^**		**Posaconazole^b^**		**Caspofungin^b^**		**Micafungin^b^**		**Anidulafungin^b^**		**5-Flucytosine^b^**		**Amphotericin B^b^**	
			**S**	**R**	**S**	**R**	**WT**	**NWT**	**WT**	**NWT**	**S**	**R**	**S**	**R**	**S**	**R**	**WT**	**NWT**	**WT**	**NWT**
Overall	3702 (100.0)	2192 (100.0)	71.2	22.2	63.8	20.3	83.1	16.9	28.3	71.7	99.0	0.7	99.0	0.8	97.6	0.6	99.0	1.0	99.8	0.2
Clinical service																				
Inpatient	3491 (94.3)	2070 (94.4)	71.4	22.1	63.8	20.1	83.1	16.9	28.5	71.5	98.9	0.7	99.0	0.8	97.5	0.6	99.0	1.0	99.8	0.2
Medical	1110 (30.0)	703 (32.1)	65.7	28.1	58.5	26.5	76.4	23.6	26.9	73.1	98.9	0.9	98.9	0.9	97.6	1.0	99.4	0.6	99.4	0.6
ICU	1092 (29.5)	618 (28.2)	74.3	19.6	67.9	17.9	87.2	12.8	31.4	68.6	98.9	0.6	99.0	0.8	96.8	0.5	99.5	0.5	99.8	0.2
Surgical	1086 (29.3)	649 (29.6)	74.2	17.9	65.4	15.4	86.7	13.3	28.4	71.6	99.2	0.3	99.4	0.5	98.3	0.3	98.2	1.8	100.0	0.0
Other	203 (5.5)	100 (4.6)	71.4	24.6	63.1	22.7	82.0	18.0	22.0	78.0	98.0	2.0	98.0	2.0	97.0	1.0	98.0	2.0	100.0	0.0
Emergency department	162 (4.4)	100 (4.6)	65.4	26.5	58.0	26.5	84.0	16.0	24.0	76.0	99.0	1.0	99.0	1.0	99.0	1.0	99.0	1.0	100.0	0.0
Outpatient	49 (1.3)	22 (1.0)	79.6	20.4	77.6	16.3	77.3	22.7	31.8	68.2	100.0	0.0	100.0	0.0	100.0	0.0	100.0	0.0	100.0	0.0
Specimen type																				
Blood	1799 (48.6)	1122 (51.2)	66.7	27.2	58.6	25.6	79.4	20.6	27.1	72.9	98.8	0.9	98.8	0.9	97.2	1.0	99.2	0.8	99.7	0.3
Non-blood samples	1903 (51.4)	1070 (48.8)	75.5	17.6	68.7	15.4	87.0	13.0	29.5	70.5	99.2	0.5	99.3	0.7	98.0	0.3	98.8	1.2	99.8	0.2
Ascitic fluid	708 (19.1)	373 (17.0)	77.5	15.8	71.6	13.8	87.7	12.3	28.7	71.3	98.9	0.5	99.2	0.8	98.1	0.3	98.7	1.3	99.7	0.3
Pus	344 (9.3)	205 (9.4)	74.4	17.7	67.4	16.0	83.4	16.6	32.7	67.3	100.0	0.0	100.0	0.0	99.0	0.0	99.5	0.5	99.5	0.5
Bile	216 (5.8)	143 (6.5)	72.7	21.3	62.5	18.1	86.7	13.3	29.4	70.6	98.6	1.4	98.6	1.4	98.6	0.7	100.0	0.0	100.0	0.0
Pleural fluid	142 (3.8)	80 (3.6)	77.5	15.5	72.5	12.7	92.5	7.5	41.3	58.8	98.8	0.0	100.0	0.0	98.8	0.0	97.5	2.5	100.0	0.0
CVC	205 (5.5)	108 (4.9)	71.2	19.5	65.9	17.6	86.1	13.9	23.1	76.9	98.1	0.9	98.1	1.9	95.4	0.9	98.1	1.9	100.0	0.0
BALF	126 (3.4)	79 (3.6)	70.6	20.6	62.7	17.5	88.6	11.4	27.8	72.2	100.0	0.0	100.0	0.0	96.2	0.0	98.7	1.3	100.0	0.0
Tissue	70 (1.9)	38 (1.7)	80.0	17.1	72.9	14.3	89.5	10.5	23.7	76.3	100.0	0.0	100.0	0.0	100.0	0.0	100.0	0.0	100.0	0.0
CSF	65 (1.8)	31 (1.4)	76.9	18.5	70.8	18.5	83.9	16.1	29.0	71.0	100.0	0.0	100.0	0.0	100.0	0.0	96.8	3.2	100.0	0.0
Other	27 (0.7)	13 (0.6)	85.2	11.1	70.4	11.1	92.3	7.7	15.4	84.6	100.0	0.0	100.0	0.0	92.3	0.0	92.3	7.7	100.0	0.0

*S, susceptible; R, resistant; WT, wild-type; NWT, non-wild-type; ICU, intensive care unit.*

*^*a*^For isolates from CHIF-NET10–18.*

*^*b*^For isolates from CHIF-NET15–18.*

Amongst the 3702 *C. tropicalis* isolates collected over 9 years, 22.2% (*n* = 823) were resistant to fluconazole, and 20.3% (*n* = 753) were resistant to voriconazole (Table 1). Moreover, 20.1% (744/3702) isolates were cross-resistant to both fluconazole and voriconazole. For 2192 isolates collected during CHIF-NET15–18, 16.9% (*n* = 370) of the isolates were of non-wild-type (NWT) phenotype to itraconazole, whilst a significantly larger proportion (*n* = 1572, 71.7%) of the isolates were of NWT phenotype to posaconazole, as per the latest CLSI ECVs (Table 1). About 16% (342/2192; 15.6%) of the isolates were resistant or of NWT phenotype to all four azoles tested.

Emerging resistance to echinocandins was observed in *C. tropicalis*, although the prevalence remained low, with 0.8% (18/2192) isolates identified in CHIF-NET15–18 being resistant to one or more echinocandin drugs, and 10 of 2192 (0.45%) isolates being resistant to all the 3 echinocandins (Table 1). In addition, 6 of 18 echinocandin resistant isolates (33.3%) were simultaneously resistant to fluconazole. Isolates of NWT phenotype to 5-flucytosine and amphotericin B were rare, with prevalence rates of 1.0% (22/2192) and 0.2% (5/2192), respectively (Table 1).

### Trend of Decreasing Azole Susceptibility Rate

**FIGURE 1:**
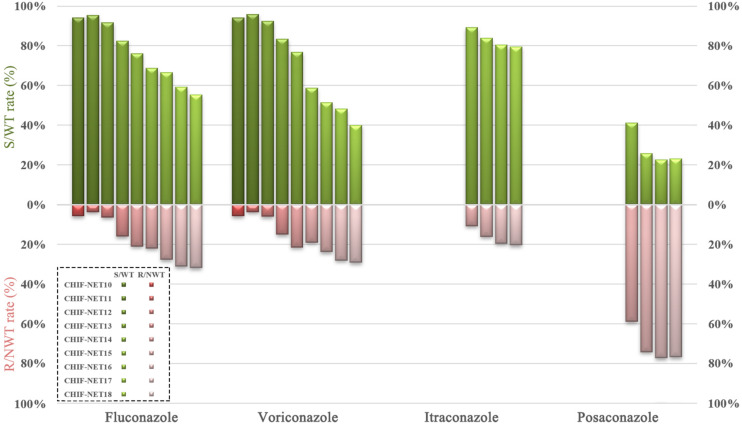
Trends of azole susceptibility in *C. tropicalis* over a 9-year surveillance. S, susceptible; R, resistant; WT, wild-type; NWT, non-wild-type.

From CHIF-NET10–18 (9 years), we have observed a tremendous decreasing trend of azole susceptibility rate for *C. tropicalis* isolates collected in China. For fluconazole, the resistance rate was only 5.7% (7/122) in the first year (CHIF-NET 10), but this rose sharply (about six times) to 31.8% (236/741) by the ninth surveillance year (*P* < 0.001) (Figure 1 and Supplementary Table 1), and the fluconazole non-susceptible rate had risen to 44.7% (331/741) (Supplementary Table 1). A similar picture was observed for voriconazole, with the resistance rate increasing from 5.7 (7/122) to 29.1% (216/741) over 9 years (*P* < 0.001) (Figure 1 and Supplementary Table 1), and furthermore, the voriconazole non-susceptible rate was even higher than that of fluconazole (444/741, 59.9%) (Supplementary Table 1). During CHIF-NET15–18, we also observed a continual increase in the proportion of NWT phenotype strains to itraconazole [from 10.7 (62/577) to 20.4% (151/741)] and posaconazole [from 58.9% (340/577) to 76.8% (569/741)] (both *P* < 0.001) (Figure 1 and Supplementary Table 1).

### Antifungal Susceptibility Across Geographic Regions

**FIGURE 2:**
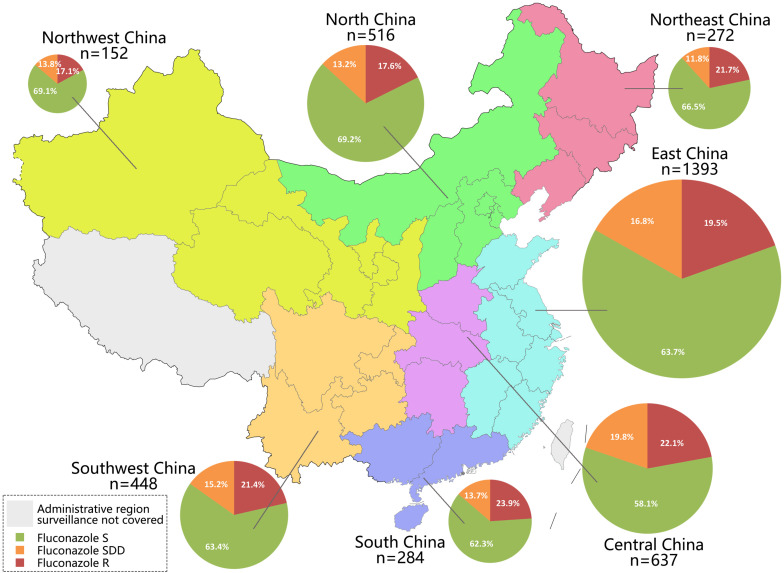
Number of isolates collected in seven administrative regions of China (labeled by different colors), and proportion of fluconazole susceptible (S), susceptible dose-dependent (SDD), and resistant (R) isolates in each region.

As China is a vast country, we further analyzed the antifungal susceptibility data to assess whether trends of declining azole susceptibility rate among *C. tropicalis* isolates was associated with geographic origins. Among seven administrative regions in China, there were 152–1393 isolates collected over nine surveillance years (Figure 2 and Supplementary Table 1). Fluconazole resistance and non-susceptible rates ranged from 18.4 to 25.0%, and from 24.3 to 32.4%, respectively (Figure 2 and Supplementary Table 1), although the differences were statistically insignificant (Chi-square test, *P* > 0.05). Voriconazole resistance rates, which ranged from 17.1 to 23.9% across different administrative regions, were also not significantly different (Figure 2 and Supplementary Table 1). However, it was observed that voriconazole non-susceptible rate in South China region (41.9%) was significantly higher than in other regions (ranged from 30.8 to 37.7%) (Bonferroni adjusted *P*-value < 0.05).

For echinocandins, it was observed that resistance to this class had emerged in six of seven administrative regions except for Northwest China, and in all regions the resistance rates were below 3% (Supplementary Table 1).

### Azole Susceptibility by Specimen Types and Clinical Services

Of 3702 *C. tropicalis* isolates collected, around half (1799/3702, 48.6%) was from blood cultures, and common non-blood specimen types included ascitic fluid (*n* = 708, 19.1%), pus (*n* = 344, 9.3%) and bile (*n* = 216, 5.8%) (Table 1). Of note, compared to isolates cultured from non-blood samples, isolates from blood cultures exhibited significantly higher azole resistance rate, which was 27.2% (489/1799) versus 17.6% (334/1903) for fluconazole (*P* < 0.001), and 25.6% (460/1799) versus 15.4% (293/1903) for voriconazole (*P* < 0.001) (Table 1). However, amongst different non-blood specimen types, there was no significant difference in azole resistance rate (*P* > 0.05). Moreover, both blood culture and non-blood culture isolates exhibited increasing resistance trends for fluconazole and voriconazole over 9 years (Supplementary Figure 2).

Overall, 94.3% of isolates collected over 9 years were from inpatient wards (3491/3702), and isolates from emergency department (4.4%, 162/3702) and outpatients (1.3%, 49/3702) were rare. Amongst strains collected in inpatient services, the proportion of isolates from medical wards, surgical wards and intensive care units (ICUs) was generally the same, ranging from 31.5 (1100/3491) to 31.1% (1086/3491) (Table 1). Statistical analysis revealed significant difference in azole resistance rates amongst medical, surgical wards, and ICUs (*P* < 0.001). *Post hoc* test indicated that the rate of azole resistance was higher in isolates from medical wards compared to those from surgical wards and ICUs. For instance, fluconazole resistance rate was 28.1% (312/1110) for isolates from medical wards, which was notably higher versus 19.6 (214/1092) and 17.9% (194/1086) for strains from ICUs and surgical wards, respectively (Bonferroni adjust *P*-value < 0.05) (Table 1).

## Discussion

Antifungal resistance has posed great challenges to clinical management of fungal infections including IC. Of note, there are significant geographic variations in the epidemiology and antifungal susceptibilities of fungal pathogens worldwide (Kullberg and Arendrup, 2015; Pappas et al., 2018). From the global SENTRY surveillance study, it was observed that *C. albicans* remained the predominant *Candida* species worldwide (overall prevalence 46.9%), and in comparison, *C. tropicalis* is the second to fourth most predominant species in different geographic regions, and is more commonly seen in Asia and Latin-America regions (14.1–17.0%) than in North America and Europe (7.5–8.0%) (Pfaller et al., 2019). However, in Asian countries like Pakistan and India, *C. tropicalis* has become the most frequently encountered *Candida* species causing candidemia (>30%) with even higher prevalence rates than *C. albicans*, while azole resistance in *C. tropicalis* remains low (<8%) in these regions (Farooqi et al., 2013; Wang et al., 2016a; Sridharan et al., 2021). Furthermore, an African country has also reported predominance of *C. tropicalis* (28.8%) in candidemia cases, but with notably high fluconazole resistance rate (31.6%) (Megri et al., 2020).

In China, *C. tropicalis* is the second to third commonest *Candida* pathogen causing IC nationwide (Liu et al., 2014; Xiao et al., 2020). But the continual rise in azole resistance rate in this species has become quite worrisome. CHIF-NET surveillance program, and a China-SCAN study (a multicenter study monitoring candidemia in ICUs in China), revealed that fluconazole resistance rate among *C. tropicalis* strains in China was steadily low (3–6%) around 2009–2012 (Wang et al., 2012; Liu et al., 2014), which was comparable to global azole resistance levels within this species (2–5% in general) (Pfaller et al., 2019). However, since then, the proportion of azole resistant *C. tropicalis* isolates in China has continued to rise. It has been reported that in 10 hospitals that consecutively participated in the first 5 years of the CHIF-NET program, fluconazole resistance rate had exceeded 20% by the fifth year (Fan et al., 2017). Further surveillance of *C. tropicalis* isolates causing candidemia revealed that >30% of strains were fluconazole-resistant by the eighth surveillance year (Xiao et al., 2020). During the same time period, there were no obvious changing trends found in fluconazole susceptibility amongst other commonly seen *Candida* species like *C. albicans*, *C. parapsilosis sensu stricto*, and *C. glabrata sensu stricto* (Song et al., 2020; Xiao et al., 2020).

In the present study, we expanded our analysis to include all IC cases. Although it was notable to find that a greater proportion of isolates from blood cultures were resistant to azoles than those from other clinical specimens (27.2 versus 17.6% for fluconazole and 25.6 versus 15.4% for voriconazole, respectively), there was also an increasing trend in the rate of resistance during the 9-year period. Furthermore, the fluconazole non-susceptible rate currently stands at over 44%, and nearly 60% of the strains were voriconazole non-susceptible nationwide in the last year. High azole resistance rates have also been reported in other recent studies in China (Song et al., 2020; Wang et al., 2020), with no obvious geographic variations in azole resistance rate amongst *C. tropicalis* strains across the country.

Molecular methods, including multilocus sequence typing (MLST), microsatellite analysis and whole genome sequencing, have been applied to investigate the phylogenetic structure of fungal pathogens. It was demonstrated that *C. tropicalis* has an extensive genetic diversity using these molecular methods, but no evidence of association between clonal population structure and geographic origins was found (Fan et al., 2017; Wu et al., 2019; O’Brien et al., 2021). However, an association between certain *C. tropicalis* phylogenetic clades, and reduced azole susceptibility, has been reported. MLST studies carried out in Thailand, Singapore, China mainland, and Taiwan, have illustrated a distinct phylogenetic clade of diploid sequence types (DSTs), including DST225, DST 376, DST 505, DST 506, DST506, DST525, DST546, etc., that are associated with azole non-susceptibility (Wang et al., 2016b, 2020; Chew et al., 2017; Tulyaprawat et al., 2020). Microsatellite analysis also revealed an association between certain genetic clusters and decreasing azole susceptibility in China (Fan et al., 2017). It is worth noting that most of the related reports on *C. tropicalis* azole resistance are from Asian countries, suggesting that Asia is probably the geographic origin of these azole non-susceptible clones. Expansion of these clones is speculated to be responsible for the fall in azole susceptibility rate in China (Fan et al., 2017), while the described diversity in DST or microsatellite molecular types, suggests continual microevolution within these clones.

Several mechanisms for azole resistance in *Candida* species have been described. *ERG11* gene mutation remains one of the most common and well-understood azole resistance mechanisms in *C. tropicalis*. Azole non-susceptible *C. tropicalis* isolates carrying substitution Y132F in Erg11p have been reported in Turkey and Asian countries (Chew et al., 2017; Fan et al., 2019; Arastehfar et al., 2020; Castanheira et al., 2020), and this key amino acid change is also responsible for reduced azole susceptibility in many other *Candida* species, including the recently discovered “superbug” *Candida auris* (Castanheira et al., 2020; Chow et al., 2020). Of note, S154F substitution in Erg11p has consistently appeared together with Y132F in *C. tropicalis* isolates from China (Jiang et al., 2013; Fan et al., 2019), but presence of S154F alone did not change azole MICs (Chen et al., 2019). There have been other Erg11p amino acid substitutions reported, such as P56S and K143R, predominantly found in fluconazole resistant *C. tropicalis* isolates in Algeria and Brazil (Xisto et al., 2017; Megri et al., 2020). Other mechanisms, including modulation of *ERG* genes and up-regulation of drug efflux pumps, also influence azole susceptibility of *C. tropicalis* (Fan et al., 2019; Arastehfar et al., 2020; Silva et al., 2020).

The emergence of echinocandin resistance among the *C. tropicalis* isolates in this study, albeit small proportion of about 0.8%, is a cause for concern as this class of antifungal drugs is highly active against most *Candida* species with minimal adverse effects, and has been recommended as first-line therapy for candidemia by Infectious Diseases Society of America since 2016 (Pappas et al., 2016). However, an increase in echinocandin resistance rate has been observed in *C. glabrata* and *C. tropicalis* in North America (Pfaller et al., 2019). Moreover, apart from the fact that echinocandin resistance has emerged in six of seven administrative regions in China, we also observed that over 30% of echinocandin resistant isolates were also resistant to azoles, which has rarely been reported from other countries.

There are several limitations in this study. Firstly, there were disparities between numbers of participating hospitals and isolates collected from different geographic regions, which may affect the accuracy of the data used for analysis. Secondly, antifungal susceptibility testing was carried out using different methods in CHIF-NET10–14 and CHIF-NET15–18, although previous studies have shown good correlation between disk diffusion and commercial Sensititre YeastOne methods to CLSI standard broth microdilution method (Pfaller et al., 2003; Espinel-Ingroff et al., 2004; Xiao et al., 2015). Moreover, CHIF-NET study remains a laboratory-based surveillance to date, primarily focused on yeasts strains causing invasive infections, and detailed clinical data, including patient management and antifungal consumption, is not systematically collected. Therefore, we are unable to determine the exact reason for the sharp decline in azole susceptibility in China, but highly speculate that it is due to azole overuse resulting in accelerated development of resistance.

In conclusion, the continual decreasing trend in the rate of azole susceptibility amongst *C. tropicalis* isolates was observed over 9 years in China. The rate of resistance to azoles was higher in isolates from blood cultures and medical wards, whilst resistance rates were statistically insignificant across geographic regions. Emergence of echinocandin- and multidrug-resistant isolates was also noted and is a worrying trend needing further scrutiny so that urgent efforts can be directed at arresting the trend.

## Members of the China Hospital Invasive Fungal Surveillance Net (CHIF-NET) Study Group

List of principal investigators in the participating hospitals from CHIF-NET study group (ranked by number of *C. tropicalis* isolates contributed): Zi-Yong Sun, Zhong-Jv Chen, Tongji Hospital, Tongji Medical College of Huazhong University of Science and Technology; Ying-Chun Xu, Meng Xiao, Peking Union Medical College Hospital, Chinese Academy of Medical Sciences; Mei Kang, Yu-Ling Xiao, West China Hospital, Sichuan University; Ya-Ning Mei, Fang Ni, Jiangsu Province Hospital; Yu-Hong Pan, Lan-Mei Gao, Fujian Medical University Union Hospital; Hui Xu, Hui Xu, First Affiliated Hospital of Zhengzhou University; Kang Liao, Peng-Hao Guo, First Affiliated Hospital, Sun Yat-sen University; Hai-Shen Kong, Qing Yang, First Affiliated Hospital, Zhejiang University School of Medicine; Wei-Ping Wang, Jinling Hospital, Nanjing University School of Medicine; Yan-Ping Luo, Li-Yan Ye, Chinese PLA General Hospital, Medical School of Chinese PLA; Hua Yu, Lin Yin, Sichuan Provincial People’s Hospital, Sichuan Academy of Medical Sciences; Da-Wen Guo, Lan-Ying Cui, First Affiliated Hospital of Harbin Medical University; Peng-Peng Liu, Hong He, Affiliated Hospital of Qingdao University; Yan Jin, Hui Fan, Shandong Provincial Hospital; Yun-Song Yu, Jie Lin, Sir Run Run Shaw Hospital; Ruo-Yu Li, Zhe Wan, Peking University First Hospital; Ling Ma, Shuai-Xian Du, Union Hospital, Tongji Medical College, Huazhong University of Science and Technology; Wen-En Liu, Yan-Ming Li, Xiangya Hospital, Central South University; Tie-Li Zhou, Qing Wu, First Affiliated Hospital of Wenzhou Medical University; Xin-Lan Hu, Ning Li, Fujian Provincial Hospital; Rong Zhang, Hong-Wei Zhou, Second Affiliated Hospital Zhejiang University School of Medicine; Yi-Min Li, Dan-Hong Su, First Affiliate Hospital of Guangzhou Medical University; Qiang-Qiang Zhang, Li Li, Huashan Hospital, Fudan University; Yun Xia, Li Yan, First Affiliated Hospital of Chongqing Medical University; Zhi-Dong Hu, Na Yue, Tianjin Medical University General Hospital; Yan Jiang, Tianjin First Central Hospital; Zhi-Yong Liu, Yu-Ting Zheng, Southwest Hospital of Army Medical University; Wei Cao, Second Xiangya Hospital of Central South University; Yun-Zhuo Chu, Fu-Shun Li, First Hospital of China Medical University; Yun Liu, Changhai Hospital; Yuan-Hong Xu, Ying Huang, First Affiliated Hospital of University of Science and Technology of China; Wei Jia, Gang Li, General Hospital of Ningxia Medical University; Huo-Xiang Lv, Qing-Feng Hu, Zhejiang Provincial People’s Hospital; Xiu-Li Xu, Xiao-Yan Chen, Air Force Medical University; Xiao-Ling Ma, Huai-Wei Lu, First Affiliated Hospital of University of Science and Technology of China; Yin-Mei Yang, Hui-Ling Chen, Guangzhou First People’s Hospital; Jian-Sheng Huang, Hui Jing, Lisui Municipal Central Hospital; Bin San, Yan Du, First Affiliated Hospital, Kunming Medical University; Hong-Jie Liang, First Affiliated Hospital of Guangxi Medical University; Bin Yang, Yu-Lan Lin, First Affiliated Hospital of Fujian Medical University; Shan-Mei Wang, Qiong Ma, Henan Provincial People’s Hospital; Hong-Mei Zhao, Li-Wen Liu, People’s Hospital of Liaoning Province; Qing Zhang, Fei Xia, Ruian People’s Hospital; Jin-Ying Wu, Mao-Li Yi, Yantai Yuhuangding Hospital; Xiang-Yang Chen, People’s Hospital of Zhengzhou; Wei-Ping Lu, Dao-Hong Zhou, Daping Hospital, Third Military Medical University; Xiao-Yan Zeng, Jing Zhang, First Affiliated Hospital of Xi’an Jiaotong University; Jing Wang, Xiao-Guang Xiao, First Affiliated Hospital of Dalian Medical University; Jia-Yin Liang, Fan-Hua Huang, Third Attached Hospital, Sun Yat-sen University; Gui-Ling Zou, Xue-Fei Du, Fourth Hospital of Harbin Medical University; Xiao-Ming Wang, Xu-Feng Ji, First Bethune Hospital of Jilin University; Yong Liu, Zhi-Jie Zhang, Shengjing Hospital of China Medical University; Yu-Xing Ni, Sheng-Yuan Zhao, Ruijin Hospital, Shanghai Jiao Tong University of Medicine; Xiu-Lan Song, First Hospital of Jiaxing; Chun-Yan Xu, Chun-Yan Xu, Taizhou Hospital of Zhejiang Province; Lin Meng, Lanzhou University Second Hospital; Xian-Feng Zhang, Ya-Lu Ren, First Affiliated Hospital of Soochow University; Jian-Hong Zhao, Hong-Lian Wei, Second Hospital of Hebei Medical University; Xue-Song Xu, Weil Li, China-Japan Union Hospital of Jilin University; Yu-Ping Wang, Mei Xu, Affiliated Hospital of Guizhou Medical University; Yun-Duo Wang, Jing Song, Dalian Municipal Central Hospital; Tian-Pen Cui, Zhi-Min Hu, WuHan No.1 Hospital; Ting-Yin Zhou, Hai-Qing Hu, Shanghai Changzheng Hospital; Xiao-Min Xu, Shan-Yan Liang, Hwa Mei Hospital, University of Chinese Academy of Sciences; Lin-Qiang Deng, Hui Chen, Jiangxi Province People’s Hospital; Xiao-Jun Sun, First Affiliated Hospital of Shandong First Medical University; Hai-Bin Wang, Jing Zhu, Fouth Medical Center of PLA General Hospital; Jian-Bang Kang, Second Hospital of Shanxi Medical University; Tie-Ying Hou, Guangdong Provincial People’s Hospital, Guangdong Academy of Medical Sciences; Ping Ji, Na Chen, First Affiliated Hospital of Xinjiang Medical University; Wen-Jun Sui, Hai-Tong Gu, Beijing Tongren Hospital, Capital Medical University; Xiao-Qin Ha, Yuan-Yuan Zhang, General Hospital of Lanzhou Military Region; Shu-Feng Wang, Hong Lu, First Hospital of Shanxi Medical University; Yi-Hai Gu, Xuan Hou, 3201 Hospital; Rong Tang, Shanghai General Hospital; Yan-Yan Guo, Fei Huang, Tangshan Gongren Hospital; Long-Hua Hu, Xiao-Yan Hu, Second Affiliated Hospital of Nanchang University; Juan Li, People’s Hospital of Xinjiang; Lian-Hua Wei, Xin Wang, Gansu Provincial Hospital; Dan Liu, Jiujiang No.1 People’s Hospital; Yan-Qiu Han, Jun-Rui Wang, Affiliated Hospital of Inner Mongolia Medical University; Yi-Hui Yao, Zhongshan Hospital, Xiamen University; Jian-Sheng Wang, Jie Wang, Hebei General Hospital; Wei Li, Qilu Hospital of Shandong University; Li-Ping Ning, 94th Hospital of Chinese PLA; Wei-Qing Song, Yu-Jie Wang, Qingdao Municipal Hospital; Liang Luan, General Hospital of Northern Theater Command.

## Data Availability Statement

The original contributions presented in the study are included in the article/Supplementary Material, further inquiries can be directed to the corresponding author/s.

## Ethics Statement

The studies involving human participants were reviewed and approved by Ethics Committee of Peking Union Medical College Hospital. Written informed consent for participation was not required for this study in accordance with the national legislation and the institutional requirements.

## Author Contributions

YW, XF, HW, and MX conceived and designed the experiments. XF, Y-NM, FN, Y-HP, L-MG, HX, H-SK, QY, W-PW, H-YX, Y-PL, L-YY, and MX performed the experiments. YW, XF, TK, and MX performed the data analysis and wrote the manuscript. All authors participated in the critical review of this manuscript.

## Conflict of Interest

The authors declare that the research was conducted in the absence of any commercial or financial relationships that could be construed as a potential conflict of interest.
