# Supplementary material for: Continual Decline in Azole Susceptibility Rates in Candida tropicalis Over a 9-Year Period in China
Source: Front Microbiol. 2021 Jul 9;12:702839. doi: 10.3389/fmicb.2021.702839 (PMC8299486; doi:10.3389/fmicb.2021.702839)
Supplement: Supplementary file 1 [file Data_Sheet_1.PDF]

Supplementary Table S1. Distribution and antifungal susceptibility of *C. tropicalis* by surveillance year and geographic regions

Characters	No. of isolates (%)		Antifungal susceptibility (%)																	
	CHIF-NET10-18	CHIF-NET15-18	Fluconazole [a]		Voriconazole [a]		Itraconazole [b]		Posaconazole [b]		Caspofungin [b]		Micafungin [b]		Anidulafungin [b]		5-Flucytosine [b]		Amphotericin B [b]	
			S	R	S	R	WT	NWT	WT	NWT	S	R	S	R	S	R	WT	NWT	WT	NWT
**Surveillance year**																				
CHIF-NET10	122 (3.3)		94.3	5.7	94.3	5.7	ND	ND	ND	ND	ND	ND	ND	ND	ND	ND	ND	ND	ND	ND
CHIF-NET11	218 (5.9)		95.4	3.7	95.9	3.7	ND	ND	ND	ND	ND	ND	ND	ND	ND	ND	ND	ND	ND	ND
CHIF-NET12	265 (7.2)		91.7	6.4	92.5	6.0	ND	ND	ND	ND	ND	ND	ND	ND	ND	ND	ND	ND	ND	ND
CHIF-NET13	411 (11.1)		82.5	16.1	83.5	15.1	ND	ND	ND	ND	ND	ND	ND	ND	ND	ND	ND	ND	ND	ND
CHIF-NET14	494 (13.3)		76.1	21.1	76.7	21.5	ND	ND	ND	ND	ND	ND	ND	ND	ND	ND	ND	ND	ND	ND
CHIF-NET15	577 (15.6)	577 (26.3)	68.8	22.0	58.8	19.1	89.3	10.7	41.1	58.9	99.1	0.3	99.0	0.7	98.1	0.5	99.3	0.7	99.8	0.2
CHIF-NET16	415 (11.2)	415 (18.9)	66.5	27.7	51.3	23.9	83.9	16.1	25.8	74.2	98.8	1.0	99.0	1.0	99.3	0.2	98.8	1.2	99.8	0.2
CHIF-NET17	459 (12.4)	459 (20.9)	59.3	31.2	48.1	28.1	80.4	19.6	22.7	77.3	98.7	0.7	98.9	0.7	98.3	0.9	99.1	0.9	100.0	0.0
CHIF-NET18	741 (20.0)	741 (33.8)	55.3	31.8	40.1	29.1	79.6	20.4	23.2	76.8	99.1	0.8	99.2	0.8	96.0	0.8	98.8	1.2	99.6	0.4
**Geographic regions**																				
East	1393 (37.6)	832 (38.0)	72.4	21.7	66.5	21.7	83.5	16.5	31.5	68.5	99.2	0.7	99.0	0.8	97.5	0.6	98.9	1.1	99.8	0.2
Central	637 (17.2)	409 (18.7)	75.2	19.8	69.2	17.6	78.7	21.3	18.1	81.9	99.5	0.2	99.8	0.2	98.5	0.2	98.3	1.7	99.5	0.5
North	516 (13.9)	266 (12.1)	72.2	21.4	63.7	19.5	80.8	19.2	24.4	75.6	98.9	0.8	99.2	0.8	97.7	0.4	100.0	0.0	100.0	0.0
Southwest	448 (12.1)	299 (13.6)	67.6	25.0	62.3	23.9	90.6	9.4	37.8	62.2	98.0	1.3	98.3	1.3	95.7	1.3	98.7	1.3	99.7	0.3
South	284 (7.7)	171 (7.8)	68.0	24.5	58.1	22.1	80.7	19.3	29.8	70.2	98.8	1.2	98.8	0.0	98.8	0.6	98.8	1.2	100.0	0.0
Northeast	272 (7.3)	114 (5.2)	75.7	18.4	69.1	17.1	85.1	14.9	30.7	69.3	97.4	0.0	97.4	2.6	97.4	1.8	100.0	0.0	100.0	0.0
Northwest	152 (4.1)	101 (4.6)	68.1	24.3	63.4	21.4	83.2	16.8	19.8	80.2	100.0	0.0	100.0	0.0	99.0	0.0	100.0	0.0	100.0	0.0

NOTE: [a] For isolates from CHIF-NET10-18; [b] For isolates from CHIF-NET15-18.

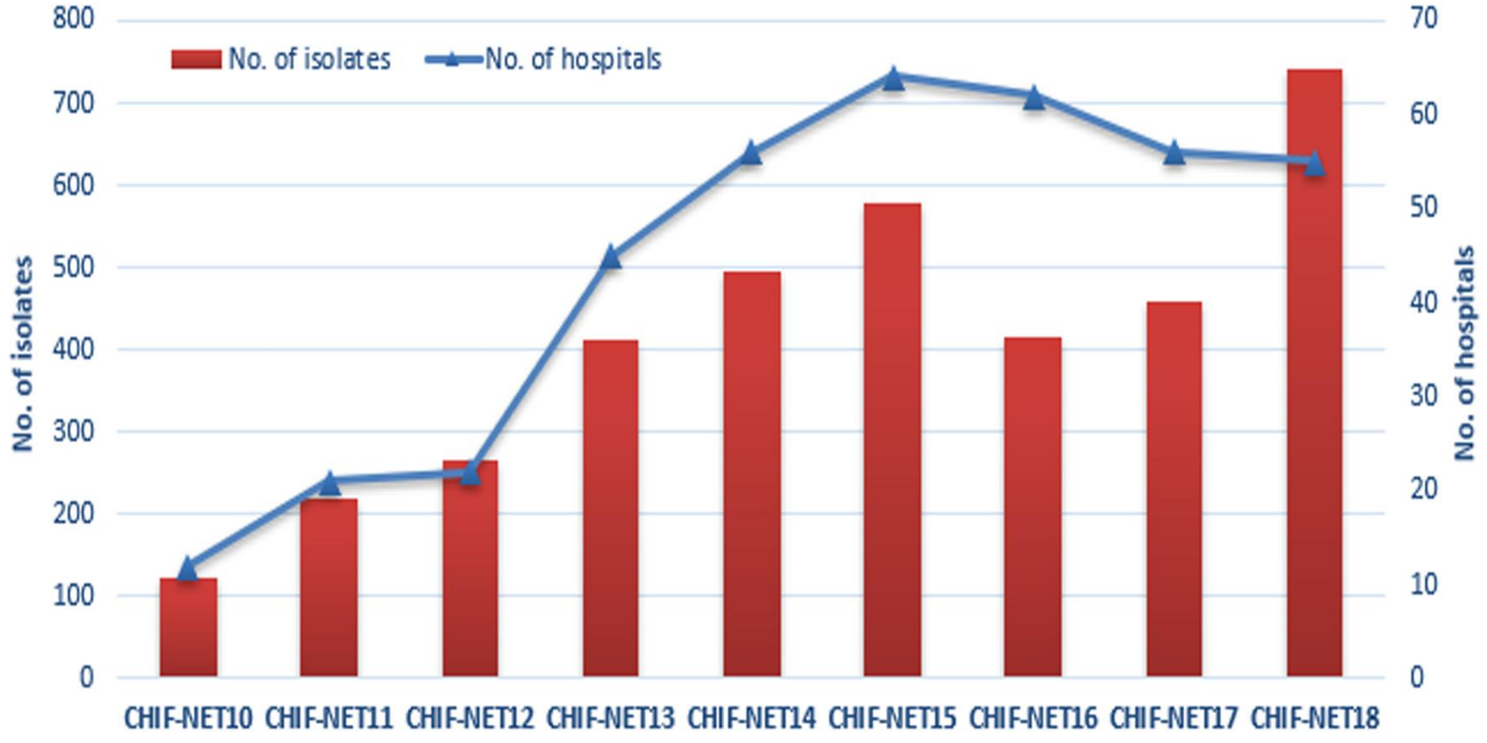

Supplementary Figure S1. number of hospitals participated and *C. tropicalis* isolates collected in each surveillance year.

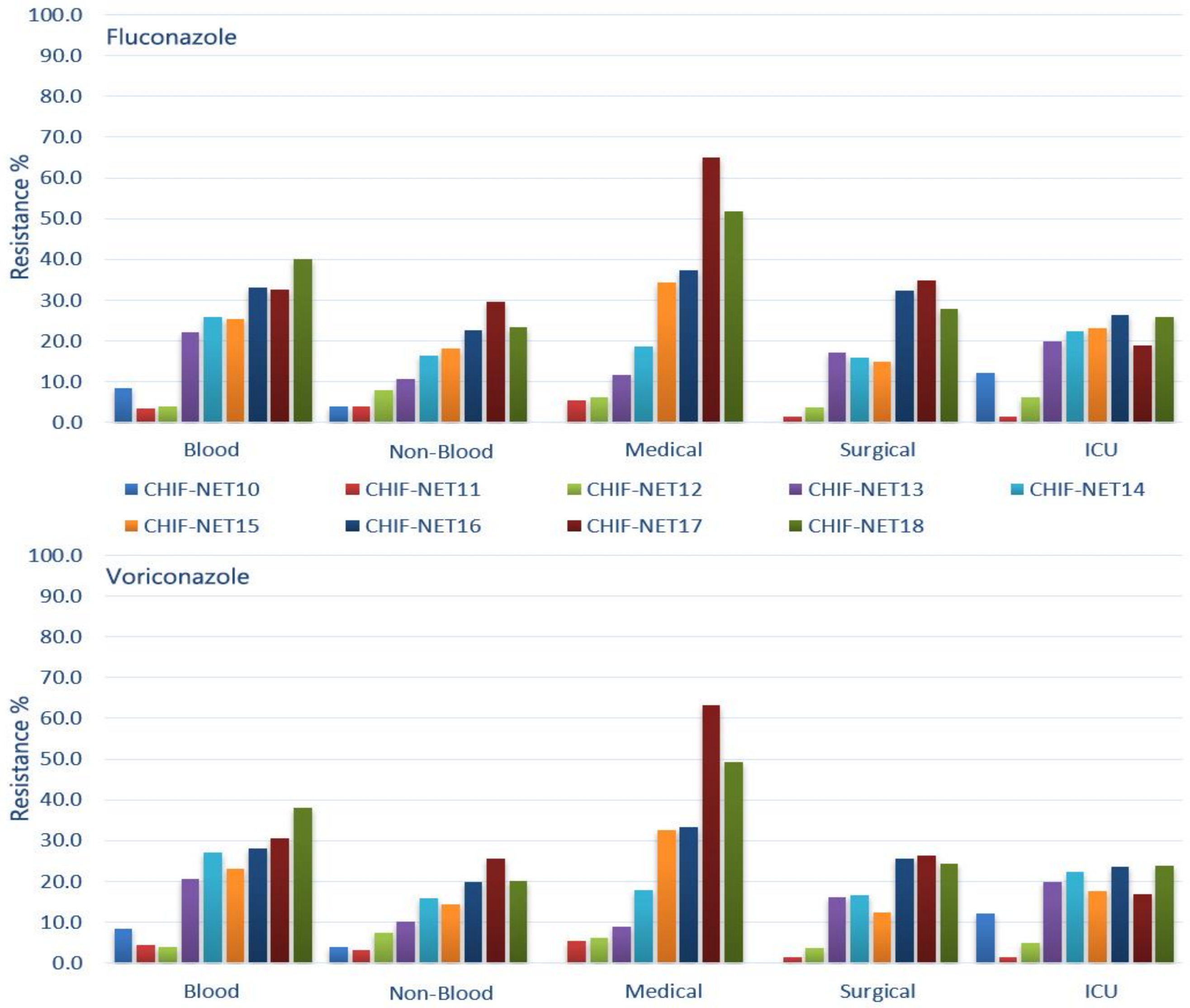

Supplementary Figure S2. Azole resistant trends amongst different specimen types and clinical wards.
